# Gene set analysis for longitudinal gene expression data

**DOI:** 10.1186/1471-2105-12-273

**Published:** 2011-07-03

**Authors:** Ke Zhang, Haiyan Wang, Arne C Bathke, Solomon W Harrar, Hans-Peter Piepho, Youping Deng

**Affiliations:** 1 School of Medicine & Health Sciences, University of North Dakota, Grand Forks, ND 58202, USA; 2 Department of Statistics, Kansas State University, Manhattan, KS 66506, USA; 3 Department of Statistics, University of Kentucky, Lexington, KY 40506, USA; 4 Department of Mathematical Sciences, University of Montana, Missoula, MT 59812, USA; 5 Institut für Kulturpflanzenzüchtung, Universität Hohenheim, D70599 Stuttgart, Germany; 6 Department of Internal Medicine, Rush University Medical Center, Chicago, IL 60612, USA

## Abstract

**Background:**

Gene set analysis (GSA) has become a successful tool to interpret gene expression profiles in terms of biological functions, molecular pathways, or genomic locations. GSA performs statistical tests for independent microarray samples at the level of gene sets rather than individual genes. Nowadays, an increasing number of microarray studies are conducted to explore the dynamic changes of gene expression in a variety of species and biological scenarios. In these longitudinal studies, gene expression is repeatedly measured over time such that a GSA needs to take into account the within-gene correlations in addition to possible between-gene correlations.

**Results:**

We provide a robust nonparametric approach to compare the expressions of longitudinally measured sets of genes under multiple treatments or experimental conditions. The limiting distributions of our statistics are derived when the number of genes goes to infinity while the number of replications can be small. When the number of genes in a gene set is small, we recommend permutation tests based on our nonparametric test statistics to achieve reliable type I error and better power while incorporating unknown correlations between and within-genes. Simulation results demonstrate that the proposed method has a greater power than other methods for various data distributions and heteroscedastic correlation structures. This method was used for an IL-2 stimulation study and significantly altered gene sets were identified.

**Conclusions:**

The simulation study and the real data application showed that the proposed gene set analysis provides a promising tool for longitudinal microarray analysis. R scripts for simulating longitudinal data and calculating the nonparametric statistics are posted on the North Dakota INBRE website http://ndinbre.org/programs/bioinformatics.php. Raw microarray data is available in Gene Expression Omnibus (National Center for Biotechnology Information) with accession number GSE6085.

## Background

Molecular biology, which is targeted at studying biological systems at a molecular level, has provided rich information of individual cellular components and their contributions to biological functions over the last 50 years. Our understanding of genes and their functions has been accelerated in the last decade by microarray experiments, which identify genes that are induced or repressed in a specific biomedical condition [1-3]. The multiplicity and heterogeneity of these gene expression profiles revealed that even a simple biological process or a molecular function in a cell requires co-operations of hundreds or even thousands of genes. Nonetheless, decoding this kind of gene interaction and networking in a biological process is hampered by the complexity of biological systems.

Instead of looking at individual genes, researchers started to interpret biological phenomena in terms of groups of genes, or gene sets. For example, Segal *et al*. (2004) mined a large number of cancer expression profiles and deduced 456 cancer-related modules (gene sets) which are selected by combining with the knowledge of transcriptional pathways and gene ontology [4]. The development of new statistical tools enables us to test whether a gene set is activated in the microarray dataset of interest. An important contribution is made by Subramanian *et al*. (2005) who proposed the gene set enrichment analysis (GSEA) to assess the significance of a set of genes. Their idea is that the genes that cooperate in a biological function have similar patterns in transcriptional levels such that the statistical power of assessing a gene set is higher than that of individual genes [5].

GSEA relies on permutation tests to identify the significant gene sets that have distinct gene expression between treatment groups. It works in three steps. First, all genes are ranked according to their statistics for the treatment effect. For example, a t-statistic can be used to compare two classes of samples. A score is assigned to each gene set using a weighted Kolmogorov-Smirnov-like statistic that sums up the ranks of the genes. Secondly, the class labels of the samples are permuted for a number of times, and gene set scores are calculated for each new label assignment. The permutation of sample labels preserves the inherent correlation between genes. Because the permutation is conducted under the null hypothesis of no treatment differences, the P value of each observed score can be determined empirically by the null score distribution. Thirdly, if more than one gene set is tested, the P values should be adjusted for multiple tests. GSEA is often applied for hundreds of gene sets, for which the false discovery rate (FDR) is recommended.

Ever since GSEA was introduced, it has drawn a wide attention from the biomedical and biostatistical communities. A number of alternative and extended versions of gene set analysis method (GSA) have been proposed in the last few years that use a variety of score systems and randomization procedures to resample data [6,7]. For instance, Efron et al. proposed a GSA method, which is based on a more powerful statistic *maxmean *to score gene sets [8]. In the case of two sample classes, *maxmean *is the maximum absolute value between the average of the positive t-statistics, and also the average of the negative t-statistics. Before permutation test, the *maxmean *score should be restandardized by centering and scaling its mean and standard deviation using randomized gene sets.

Despite their enormous success, all these aforementioned GSA methods have limited applications in microarray samples with dependence. A permutation test has to rely on the assumption of sample independence. This assumption presents a barrier to extend GSA to the fast-growing area of longitudinal microarray experiments, which repeatedly profiles the gene expression of a same object over time. Longitudinal microarray experiments allow researchers to investigate dynamic behavior of biological processes, such as cell cycles, cell proliferation, oncogenosis, and apoptosis. The temporal component is an inherent part of the study. Such time course experiments pose novel challenges for statistical analyses because effective methods have to take into account both a large number of genes and within-gene correlations. Most of the analyses in literature carry out repeated measures analysis of individual genes followed by FDR control [9-12].

It is desirable to apply repeated measures analysis methods, such as a linear mixed effects model (LME) or generalized estimating equations (GEE), to gene sets. Tsai and Qu (2008) assessed subsets of genes by applying a non-parametric time-varying coefficient model [13]. The within-gene correlation was taken into account by the quadratic inference function (QIF) that is derived from GEE. Both LME and GEE achieve their asymptotic distributions when the number of replications goes to ∞. However, the large sample size assumption is usually not applicable due to the high cost of microarray experiments. Rather, there is often a relatively large number of genes in a gene set compared to the sample size, a curse of dimensionality problem. An effective GSA method should also be robust against deviation from the normal distribution because gene expression data may be largely skewed, and the normal or log-normal distribution does not provide a close fit to the data [14,15]. Furthermore, to allow variability between genes, heteroscedastic correlation structures should be assumed for different genes.

In this paper we propose a GSA method for assessing the expression patterns of gene sets from longitudinal microarray data. The method employs a couple of novel nonparametric statistics that work for small sample size as long as we maintain a relative large number of genes in a set (large p, small n). The method is robust with respect to non-normality and heteroscedastic correlation structures. To ensure extensive application, unbalanced designs are allowed in our model. For example, unbalanced data may occur when the data are pooled from different versions or manufacturers of arrays.

The genes in a signal transduction pathway are often highly correlated in that the expression of one gene is regulated by the other gene in this pathway. To ensure an unbiased analysis, we need to take into account the correlation among genes. Permutation method has been widely used in GSA to provide a robust test that preserves between-genes correlations. For example, Tsai and Chen (2009) used permutation test with the Wilks' Λ statistic for their multivariate analysis of GSA [16]. To take into account the correlations among genes within a gene set, we also present a permutation-based test for our proposed statistics.

The outline of this paper is as follows. Our main results are presented in section Results and Discussion. In subsection Model and Hypotheses, we describe the model and assumptions. In the subsection of Simulation study, we present the simulation results of type I error estimates and power analysis for our proposed methods. In subsection Results on real data, we describe an application of our method to a recent longitudinal microarray study in which the gene expression profiles of murine T cells in the presence or absence of interleukin-2 (IL-2) were repeatedly collected. A number of functional gene sets were tested to investigate IL-2 signaling over time. The test statistics and their asymptotic results for a large number of genes but small replications are provided in subsection Test statistics of section Methods. Subsection Permutation tests described the permutation-based test with our proposed nonparametric statistics. Finally, we provide mathematical proof for the asymptotic results of our test statistics in Appendix.

## Results and Discussion

### Model and hypotheses

In a longitudinal design for microarray studies, global transcriptional levels of each object were repeatedly measured at multiple time points under various conditions, such as different drug doses, genotypes, and chemical environments. Our goal is to find whether the transcription levels of a set of genes show a dynamic pattern that differs between conditions. We enumerate all the conditions using *i *= 1,..., *I *and refer them as treatments. If the number of genes in a gene set is relatively small compared to the number of sample replications, the methods for repeated measures analysis, such as LME and GEE, are able to test the variation among treatments under certain distributional assumptions. Both LME and GEE provide efficient model parameter estimates when the assumed covariance matrices can be estimated consistently. However, when the number of genes plus the number of time points is much larger than the number of replications, consistent estimates of the large covariance matrices are no longer available, especially if multiple large covariance matrices need to be estimated when empirical evidence suggests heteroscedasticity is present. We will focus our e ort on the latter case.

For a gene set, let **X**_*ikl *_= (*X*_*i*1*kl*_,..., *X*_*iJkl*_)' be the transcriptional levels of the *k^th ^*gene (or probe) of the *l^th ^*replicate in treatment *i*, where *k *= 1,..., *K*, *l *= 1,..., *n_ik_*, and *i *= 1,..., *I*. The expression of this gene is measured at J time points with subscript *j *to enumerate the *j^th ^*repeated measurement. Denote *μ_ik _*= *E*(**X**_*ikl*_) and Σ_*ik *_= Var(**X**_*ikl*_) = (*σ*_*i*, *k*, *jj'*_)_*J*×*J *_to be the gene specific mean and covariance matrix. Each individual gene has its own transcriptional activity, therefore, each gene has its unique correlation structure. The heteroscedastic covariances for different treatments and different genes allow us to take into account of the different mechanisms that different genes respond to a treatment. This is more realistic than assuming a common covariance matrix in that many of the genes are not responsive to a specific stimulus while the responsive genes could exhibit different temporal dependence. An example is that a stimulus specific regulator gene or transcription factor tends to be activated at the early stage of the stimulus and the downstream genes of the regulator will respond at a later stage. We leave the joint distribution of **X***_ikl _*unspecified and assume the observations from different treatments or replicates are independent.

Let  be the mean expression profile for the *i^th ^*treatment. Let ***α ***be the *I *× *J *matrix with *i^th ^*row being  The hypothesis of no effect for the contrast of the treatments can be stated as(0.1)

where **L**_1 _is a *p *× *I *contrast matrix with full row rank, **1***_J _*is the *J*-dimensional vector of ones, and **0***_p _*is a *p*-dimensional vector of zeros. The contrast matrix is convenient to assess the effect of a specific treatment factor if the treatment consists of multiple factors. Typical contrast matrix for a single treatment factor with *I *levels is an (*I *- 1) by *I *matrix **L**_1 _= (**1**_*I*-1_| - diag(*I *- 1)), where the first column is **1**_*I*-1_, a column vector of ones, and the remaining columns are -*diag*(*I *- 1), the negative of the identity matrix of dimension (*I *- 1). For *I *= 3, the above **L**_1 _is

This particular contrast matrix basically specifies that all the treatment means some treatments averaged over the whole time period and over all genes are identical. Differences could arise if the mRNA transcriptions of some genes are activated or inhibited by the treatment. Genes could have distinct expression trends over time.

The hypothesis of no effect for a contrast among the treatment by time interactions can be expressed as(0.2)

where *Vec*() function transforms a matrix into a vector by concatenating all columns, **P***_I _*is the projection matrix , and **L**_2 _is a *q *× (*IJ*) contrast matrix with full row rank. An example of the contrast matrix is the Kronecker product *M_I _*⊗*M_J _*that specifies that all interactions are zero, where *M_I _*= (**1**_*I *-1_| - diag(*I *- 1)). For example, with *I *= 3, *J *= 4, the Kronecker product contrast matrix for interaction effect is

We present a summary of notations that are used in the rest of the manuscript. Denote , and

We consider a couple of novel nonparametric statistics for hypotheses testing. A linear mixed effects model (LME) and generalized estimating equations (GEE) are often used for testing hypotheses (0.1) and (0.2) by assuming an appropriate correlation structure. The statistics for both LME and GEE achieve their asymptotic distributions when the number of samples goes to infinity. Thus, theoretically LME and GEE are not suited to large p, small n problems such as microarray data. This motivated us to propose new statistics that converge to their limiting distributions when the number of genes goes to infinity. The statistics should be robust for non-normal distributions, heteroscedastic correlation structures, and unbalanced experiment designs. Two novel Wald statistics are proposed for null hypotheses (0.1) and (0.2) in the method section. Their asymptoticity is proved in Appendix.

### Simulation study

This section will present our simulation study to evaluate the proposed nonparametric test statistics (NP) in various settings. First, we calculate the estimated type I error rate at level 0.05 for our nonparametric statistics. The type I error will be examined for samples generated from normal, exponential, Poisson and Cauchy distributions after introducing within-subject correlations. Second, we will compare the power of the NP statistics with linear mixed-effects model (LME) and generalized estimating equations (GEE). The type I error and the power analysis are used to validate our NP statistics. Thirdly, we will calculate the estimated type I error and power of the permutation test with our statistics for correlated genes and compare the results with GEE on data from normal, exponential, and Poisson distributions. All calculations and simulations were carried out with R programming and the results were based on 1000 iterations. The LME and GEE methods were implemented by using *gls *and *geese *functions from R packages *nlme *and *geepack*, respectively ([17,18]).

#### (a) Type I error rate analysis based on asymptotic distribution with simulated data

In this section, we evaluate the specificity of our proposed test (NP) based on type I error rates for simulated data from various distributions. The number of time points per gene we simulated is either 2 or 5. As balanced design is only a special form of unbalanced design, here we only consider unbalanced design in that four fifths of genes having 4 replications and the remaining one fifth of genes having 6 replications. First, we examined the proposed test statistic for no gene expression variations across treatments. A data matrix **X **of *n *rows and *J *columns were randomly generated with each row representing observations from the same gene over J time points. The n is the sum of the number of replications for all genes across all treatment groups. The rows were generated from identical distribution such that the null hypothesis of no expression changes across treatments is satisfied. To allow a wide variety of data types, we use normal, exponential, Poisson, and Cauchy distributions to generate random samples. For normal, exponential, and Poisson distributions, the mean of random data was set to 2. The normal distribution was given a standard deviation of 1. The Cauchy distribution had a location parameter of 0, and a scale parameter of 1. Unstructured within-gene correlations were then generated from a uniform distribution on (0, 0.5).

Identical unit variance is used for data under the null hypotheses. We used the Cholesky decomposition (via R function *chol*) to produce the lower half triangular matrix **h **for the covariance matrix Σ. Thus the data matrix *Y *= **Xh **has the desired covariance structure and it is used for subsequent data analysis. The matrix Y had equal means across rows. However, at different time points (across columns), the values from the same gene could vary.

Table 1 gives the estimated type I error rates for data with unstructured correlation using the asymptotic distribution of the test statistic for treatment. For normal, exponential, and Poisson distributions, the error rates for at least 5 genes and 2 or 5 time points were in high agreement with the expected level *α *= 0.05. The error rate for Cauchy distribution failed to converge to 0.05 as the number of genes increases. This happens because the test requires finite fourth central moments while Cauchy distribution does not have finite moments.

**Table 1:** Estimated Type I errors for the test of no treatment effect based on asymptotic distribution

#time.points	#genes	normal	exponential	Poisson	Cauchy
2	5	0.060	0.053	0.063	0.021
	10	0.047	0.052	0.048	0.024
	20	0.053	0.046	0.054	0.026
	30	0.048	0.063	0.058	0.019
	40	0.044	0.052	0.053	0.021
	50	0.043	0.052	0.057	0.020
	100	0.040	0.050	0.042	0.020

5	5	0.056	0.052	0.059	0.032
	10	0.053	0.055	0.057	0.025
	20	0.047	0.045	0.066	0.020
	30	0.060	0.058	0.050	0.014
	40	0.050	0.049	0.047	0.018
	50	0.044	0.041	0.041	0.016
	100	0.062	0.047	0.050	0.023

The data from the same gene have unstructured correlation.

The next test was concerned with the interaction of treatment and time effect. Under the null hypothesis of no interaction, we generated random data as follows. Given the value *y_ij _*for probe i at the *j^th ^*time point, the random observation at the (*j *+ 1)*^th ^*time point can be obtained by(0.3)

where *ε*_*ij *_is a random variable with mean 2(1 - *ρ*). Thus the mean of *X*_*i*, *j*+1 _is 2, which is the same as that of *X*_*ij*_. For the Poisson distribution, we first generated the mean values with the iterative algorithm (0.3), and then used the means to generate random integer numbers. An unstructured correlation was introduced to the repeated measures for each gene similarly as was generated for the test of no treatment effects. The type I error rates at *α *level 0.05 were shown in Table 2. Normal, exponential, and Poisson distributions had error rates close to 0*:*05 when the number of genes was above 50. Cauchy distribution did not converge to 0.05.

**Table 2:** Estimated type I error of the test of no treatment by time interaction at 0

#genes	normal	exponential	Poisson	Cauchy
5	0.087	0.103	0.099	0.046
10	0.074	0.082	0.064	0.035
20	0.061	0.063	0.050	0.024
30	0.070	0.071	0.063	0.019
40	0.071	0.060	0.065	0.019
50	0.064	0.052	0.056	0.011
100	0.037	0.051	0.048	0.012
200	0.043	0.050	0.052	0.018
500	0.048	0.040	0.051	0.022
1000	0.057	0.046	0.048	0.013

The data from the same gene followed unstructured correlation. For each simulation, there are two time points.

#### (b) Power analysis based on asymptotic distribution with simulated data

To evaluate the proposed NP statistics, we calculated the estimated power curves for three methods, NP, LME and GEE. Data were simulated for 4 treatment groups and 3 replicates. As shown in Tables 1 and 2, the number of genes being 50 or above achieves expected error rates. Therefore, we used 50 genes for all of the power analysis in this subsection. Each gene was repeatedly measured at 4 time points. Random data were generated in the similar way as for type I error simulation study. Log-normal distribution was assumed so that the data were first generated by a normal distribution and were then taken exponential transformation. An unstructured correlation was introduced between time points for each gene as described in the simulation study subsection.

For LME and GEE, gene expression levels were modeled as the response variables with treatment group and time as fixed effects. The variable subject, which provides measurements for all genes at all time points, are modeled as a random effect. Unstructured correlation structure cannot be estimated in LME and GEE model fitting due to the number of replications being small. In this part of the simulation, compound symmetry correlation structure was assumed for LME and working independence correlation structure was used for GEE.

**Figure 1:**
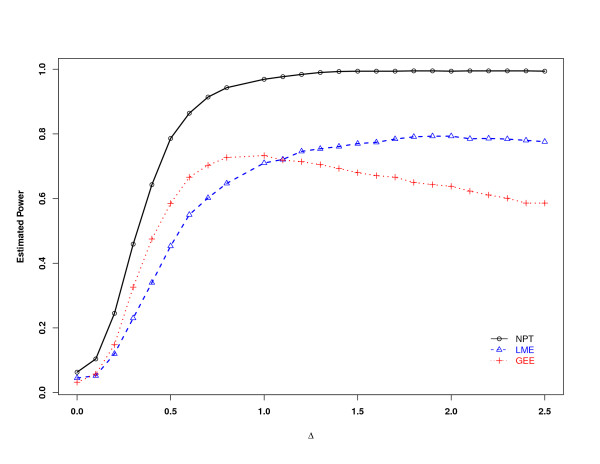
**The power curve of NP statistic based on the asymptotic distribution compared to LME and GEE**. The empirical powers of the NP statistics for testing of no treatment effect based on the asymptotic distribution compared to LME and GEE are given here. The powers were estimated at level 0.05. Δ is the log-scale mean difference between successive treatment groups.

First, we conducted a power analysis for the treatment effect. The means of the normal distributions are different between the treatment groups under alternative hypothesis, and the standard deviation of the normal distribution for each gene is randomly generated by a uniform distribution in (0, 3). The mean differences Δ between groups range from 0 to 2.5 to generate the power curves. Thus in each experiment, the logarithm of the mean of treatment group 2 is Δ higher than that of group 1, and that of group 3 is Δ higher than group 2, and so on. The three power curves for NP, LME, and GEE were shown in Figure 1. NP outperformed GEE and NP for all Δ. When Δ = 0.7, NP has 91% power, whereas LME has 60% power and GEE has 70% power.

Next, we conducted power simulation analysis for the test of no treatment and time interaction. The results were similar to that for the treatment effect. So we do not present the results here.

#### (c) Type I error and Power analyses for the permutation test

We further conducted simulation study for the permutation test with our NP statistics by generating random data that had both within-gene correlation over time and between-gene correlation within a gene set.

**Figure 2:**
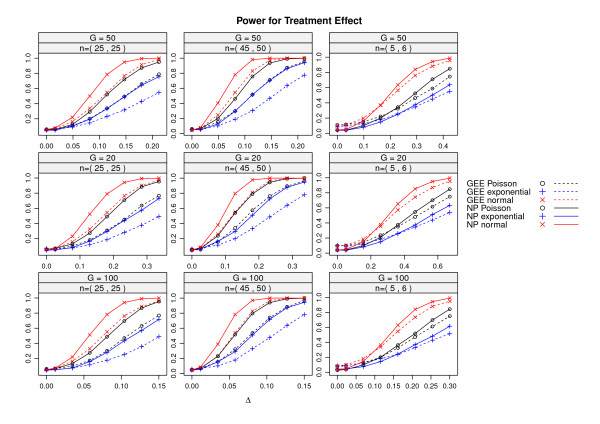
**Power comparisons for the permutation test of no treatment effect compared with GEE**. The power curves for using permutation tests for treatment effect are given here. The powers were estimated at level 0.05. *G *is the number of genes, *n *is the number of replicates in the two treatment groups, and Δ is the mean difference between the treatment groups.

Random data were generated for two treatment groups with three time points. In order to show the effects of sample size on the power, the number of replicates for a group varied from 5 to 50. Random data were generated in the same way as for power analysis of NP statistics described earlier except that an AR(1) correlation structure with correlation coefficient 0.5 was introduced to gene-gene relationship. Gene sets with 20, 50 and 100 genes were generated following normal, exponential and Poisson distributions. Since linear mixed effects model is not valid for exponential or Poisson distributions, we compare the permutation-based NP statistics with GEE. For this part of the simulation, gene expression levels were modeled as the response variables while fixed effects of treatment, time, treatment by time interaction, and gene index are included in the GEE model. The variable subject is modeled as a random effect and AR(1) correlation structure was assumed for GEE. The type I error estimates are reported in Table 3 and power estimates are given in Figure 2 as the mean differences Δ between the two treatment groups increases. It is clear from Table 3 that GEE has inflated type I error when the number of replications in each treatment group is small. The permutation test on our NP statistic has very reliable type I error rate. The result of power comparison in Figure 2 shows that the permutation test with our NP test statistic consistently has higher power in all simulation settings. This happens because the NP test statistics are particularly suitable for large p, small n settings. GEE has lower power even though specification of AR(1) structure for GEE gives some advantage to it. In real data analysis, exploring and finding the correct correlation structure for GEE is itself a challenge. The differences in performance seem to be less evident when sample sizes are small (the last column of the plots in Figure 2). However, we remark that in this case the power of GEE was most likely overestimated because of the type I error inflation (see Table 3). As the number of genes increases, the powers of both permutation NP test and GEE increase. They both show better performance for normally distributed data than data from exponential and Poisson distributions due to the skewness of exponential distribution and more variations associated with Poisson distribution than the normal data we generated.

**Table 3:** Estimated type I errors for the permutation test of no treatment effect compared to GEE

distribution	n1	n2	G	permutation NP	GEE
Normal	5	6	20	0.041	0.097
	25	25	20	0.055	0.058
	45	50	20	0.047	0.056
	
	5	6	50	0.045	0.105
	25	25	50	0.058	0.053
	45	50	50	0.046	0.044
	
	5	6	100	0.033	0.087
	25	25	100	0.053	0.058
	45	50	100	0.047	0.045

Poisson	5	6	20	0.040	0.096
	25	25	20	0.058	0.055
	45	50	20	0.058	0.049
	
	5	6	50	0.041	0.109
	25	25	50	0.058	0.061
	45	50	50	0.052	0.062
	
	5	6	100	0.028	0.075
	25	25	100	0.053	0.063
	45	50	100	0.050	0.048

Exponential	5	6	20	0.040	0.101
	25	25	20	0.046	0.070
	45	50	20	0.047	0.062
	
	5	6	50	0.041	0.083
	25	25	50	0.048	0.056
	45	50	50	0.052	0.051
	
	5	6	100	0.041	0.087
	25	25	100	0.046	0.053
	45	50	100	0.044	0.059

The data from different genes and repeated measurements from the same gene have AR(1) correlation with correlation coefficient 0.5. The n1 and n2 are the sample sizes for treatment groups 1 and 2, respectively. G is the number of genes in the gene set. The estimate is at 0.05 level.

### Results on real data

We apply the proposed method to a recent time course microarray study of mouse immune response. Cytotoxic T lymphocyte (T cells) plays a key role in cell-mediated immune response. They destroy virally infected cells, tumor cells, and other disease cells. The fast immune response to a foreign antigen relies on rapid activation and proliferation of T cells that are stimulated by a cytokine molecule, Interleukin-2 (IL-2) [19]. The gene expression profiles with IL-2 stimulation have identified approximately 3000 IL-2-regulated genes in human T cells [2,20-23]. A time course microarray study was carried out in Sandia National Laboratories to investigate activated genes by IL-2 during T cell proliferation and differentiation [24]. The murine T cell line CTLL-2 was cultured in the presence or absence (control) of IL-2 stimulation. Each treatment group has 3 independent cell cultures. For each culture, cells were harvested at 2 time points, 4 h and 8 h for microarray processing with Affymetrix Mouse Genome 430 2.0 Array. The light intensities of gene expressions were log-transformed and quantile-normalized prior to be analyzed by the proposed gene set method [25].

**Figure 3:**
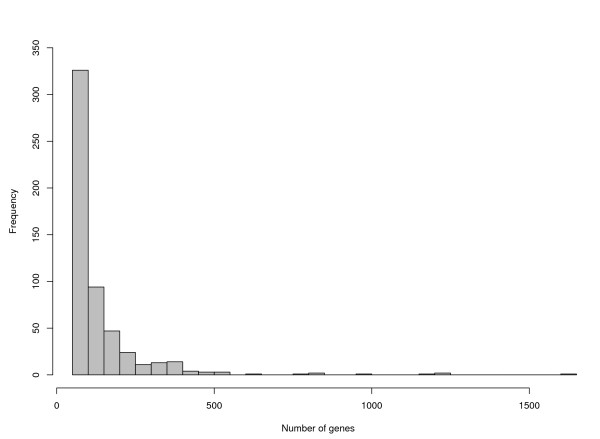
**The distribution of the gene set sizes**. The histogram showed the distribution of the size of the 548 gene sets used for data analysis.

We used the C2 collection of gene sets from the Molecular Signature Database (MSigDB) of Broad Institute. C2 collection is curated from various sources such as online pathway database, biomedical literature, and knowledge of domain experts [26]. The collection contains 1892 gene sets. Since our previous simulation studies showed that at least 50 genes are required for a gene set to achieve sufficient statistical power and appropriate type I error rate, 548 sets out of 1892 gene sets were selected that consist at least 50 genes. The distribution of the number of genes from the 548 gene sets was shown in Figure 3. In order to identify the gene sets that are regulated by IL-2, we used NP to test for the interactions of treatment and time, and the main effect of IL-2 treatment. The P value of each gene set was converted to false discovery rate (FDR) with R package *fdrtool *[27,28]. With a FDR threshold at 5%, 285 gene sets showed significant treatment×time interaction, whose biological implications need to be further investigated. Of the remaining 263 gene sets, 20 sets were identified to be significantly differentially expressed by the treatment effect test. Thus, totally 283 gene sets are responsible to IL-2. The 20 selected gene sets for the treatment effect were reported in Table 4. There were totally 1,760 distinct genes involved in the 20 gene sets.

**Table 4:** The IL-2 regulated gene sets.

Gene Set	FDR
Ross cbf	0.020
Peart histone up	0.047
Rome insulin 2f up	0.038
Hivnefpathway	0.025
Cell adhesion	0.041
Haddad hsc cd7 up	0.010
Flechner kidney transplant rejection pbl up	0.009
Shepard pos reg of cell proliferation	0.029
Haddad cd45cd7 plus vs minus up	0.010
Hsiao liver specific genes	0.031
Takeda nup8 hoxa9 3d up	0.030
Cromer hypopharyngeal met vs non dn	0.028
Vanasse bcl2 targets	0.006
Gamma unique fibro dn	0.018
Tnfalpha adip dn	0.026
Gn camp granulosa dn	0.041
Aged mouse neocortex up	0.026
Adip diff up	0.006
Hsa04370 vegf signaling pathway	0.016
Hsa04520 adherens junction	0.008

T lymphocyte activation by IL-2 culminates many cellular processes, including blastogenesis, cell cycle progression, DNA replication and Mitosis [2]. Many of the selected gene sets are known to participate in these complicated biological functions. The gene set, VANASSE BCL2 TARGETS, consists of genes that are differentially expressed in murine CD19+ B cells overexpressing Bcl-2, a key gene regulating apoptosis. This confirms the anti-apoptotic effects of IL-2 that proliferate T cells [29]. The other gene sets that have similar effects on cell proliferation and aging are SHEPARD POS REG OF CELL PROLIFERATION, GAMMA UNIQUE FIBRO DN, and AGED MOUSE NEOCORTEX UP. Some selected gene sets, such as FLECHNER KIDNEY TRANSPLANT REJECTION PBL UP and HSIAO LIVER SPECIFIC GENES, are involved in the immune response of T cell. The gene sets, HADDAD HSC CD7 UP and HADDAD CD45CD7 PLUS VS MINUS UP, are involved in T cell development. The gene sets, CELL ADHESION and HSA04520 ADHERENS JUNCTION, are responsible to the interaction of T cell with foreign cell, the core function of T cell mediated cytotoxicity. Insulin 2F related gene set, ROME INSULIN 2F UP, plays multiple roles in many gene regulating pathways including cell proliferation. The gene set HSA04370 VEGF SIGNALING PATHWAY plays a role in tumor agiogenesis. The relationship of these gene sets with IL-2 stimulation is worth further investigation.

## Conclusions

With the fast advancement of high throughput genomics technology and increased complexity of array experimental design, researchers need robust statistical tools to decipher the code of sophisticated gene-gene interaction and networking during biological processes. Gene set analysis has served as a useful tool to identify functional gene sets in recent years. To apply GSA to correlated microarray samples such as longitudinal studies, we developed a couple of novel nonparametric statistics for testing gene set variation. The proposed GSA methods assess the effects of main treatment and treatment by time interactions for a set of genes measured in longitudinal microarrays. Heteroscedastic covariance structures are assumed for a realistic modeling of complicated microarray data. The limiting distributions of the proposed test statistics were derived under the asymptotic setting of a large number of genes and small number of replications. When a gene set contains only a small number of genes, permutation test based on the proposed NP statistics has excellent power compared to GEE in our simulation study. The proposed tests were applied to a collection of gene sets from the Molecular Signature Database (MSigDB) of Broad Institute and identified a number of gene sets that are responsive to IL-2 stimulation.

## Methods

### Test statistics

#### (a) Heteroscedastic test of no treatment effect

To test *H*_0_(treatment), we consider a Wald-type test statistic:(0.4)

where , and , with

*W_A _*converges to a Chi-square distribution when the number of genes goes to infinity (see Appendix). The degrees of freedom of the limiting distribution is the same as the rank of matrix *L*_1_.

#### (b) Heteroscedastic test of no treatment and time interaction effect

The test statistic is for no contrast effect among the interactions of treatment and time is given by(0.5)

where , and  is the estimated covariance matrix for **D***_AB_*. The estimated covariance of  and is given at the ((*i *- 1)*J *+ *j*)^th ^row and ((*i*_1 _- 1)*J *+ *j*_1_)^th ^column of . If *i *≠ *i*_1_, the values is zero. If *i *= *i*_1_, the value is given by

*W_AB _*also converges to a Chi-square distribution when the number of genes goes to infinity (see Appendix). The degrees of freedom for the Chi-square distribution is the same as the rank of matrix *L*_2_.

### Permutation tests

The nonparametric statistics given in (0.4 and 0.5) take into account the within-gene correlations among multiple time points. The correlations among genes within a gene set are unknown. We are not able to incorporate them into our statistics unless the genes are ordered in a manner such that the correlations between genes diminishes with a certain rate as their distance increases. It is unrealistic to make such an assumption for a gene set whose member genes have no known ordering. Furthermore, it is possible that all genes in a gene set are highly correlated. For example, if gene A is a transcription factor and the other genes in the gene set are its downstream genes regulated by A in a pathway, all genes will have high correlations. Failure of incorporating between-genes correlations would bias our statistics.

We use a permutation-based test with the proposed nonparametric statistics to avoid bias. Specifically, we performed 400 permutations for the treatment group labels of the subjects. For each permutation, we randomly assign *n_i _*subjects with measurements from all genes at all time points to have group label *i*, where *i *= 1,.., *I*. We do not permute the genes or time points to keep their original correlations. The proposed statistics are calculated for a given gene set for each permuted sample. All statistics are then ranked, and the percentage of the statistics greater than that from the raw data gives the P value. It is interesting to note that the asymptotic distributions of our test statistics are only applicable when the number of genes is large. The permutation tests can be applied even when the number of genes is small.

## Appendix

### Asymptotic distribution of the NP test statistics

**Theorem 0.1 ***For testing H*_0_(*treatment*)*, let W_A _be the statistic given in (0.4). If X_ijkl _has a finite fourth central moment, then under H*_0_(*treatment*), 

**Proof of Theorem 0.1**: Under *H*_0_(treatment), *E*[*LD_A_*] = **0**. Hence, we have **L**_1_**D***_A _*= **L**_1_(**D***_A _***- ***E*[**D***_A_*]).

Let V*_A _*= *Var*[**D**_*A*_] = *diag*(*η*_*A*1_,..., *η*_*AI*_), where

Because of the independence of , the result will follow from the Continuous Mapping and Slutsky's Theorem if we can show that as K → ∞,(0.6)(0.7)

It is easily seen that (0.6) and (0.7) is true since Lyapounov condition is satisfied with the finite fourth central moment condition:

The convergence of (0.7) can be shown by Markov weak law of large number. Note that  since the sample covariance is an unbiased estimate of the covariance. Write

Then . The Markov condition will be satisfied if

It is sufficient to show that  is finite. By Hölder's inequality,

for fixed J and *n_ik_*. The finite bound is obtained because the first four moments of *X_ijkl _*exist. This completes the proof.

**Theorem 0.2 ***For testing H*_0_(*interaction*)*, let W_AB _be the statistic given in (0.5). If X_ijkl _has a finite fourth central moment, then under H*_0_(*interaction*), .

**Proof of Theorem 0.2: **Under *H*_0_(interaction), *E*[**L**_2_**D***_AB_*] = **0**, then **L**_2_**D***_AB _*= **L**_2_(**D***_AB _*- *E*[**D***_AB_*]). Let *V_AB _*= *Var*[*D_AB_*]. The result will follow with the Continuous Mapping and Slutsky's Theorems, by showing , where . It is sufficient to show that for any finite constant **a **= (*a*_11_, *a*_12_,..., *a_ij_*,..., *a_IJ _*)',(0.8)

where the limit of exists since it is a nonnegative quadratic form and  converges due to the bounded nature of *n_ik_*. The asymptotic normality in (0.8) can be shown by Lyapounov's Theorem. Write

Note that

where the inequalities follow from Hölder's inequality, and the last equality holds due to the finite moment condition. This completes the proof.

## Competing interests

The authors declare that they have no competing interests.

## Authors' contributions

KZ and HW developed the methods, wrote the code, performed the simulation and analysis and drafted the manuscript. AB, SH, HP and YD contributed ideas and wrote the manuscript with valuable discussions. All authors have read and approved the final manuscript.
